# Anomalous strain effect on the thermal conductivity of low-buckled two-dimensional silicene

**DOI:** 10.1093/nsr/nwaa220

**Published:** 2020-08-31

**Authors:** Bin Ding, Xiaoyan Li, Wuxing Zhou, Gang Zhang, Huajian Gao

**Affiliations:** Institute of High Performance Computing, A*STAR, Singapore 138632, Singapore; Centre for Advanced Mechanics and Materials, Applied Mechanics Laboratory, Department of Engineering Mechanics, Tsinghua University, Beijing 100084, China; School of Materials Science and Engineering & Hunan Provincial Key Laboratory of Advanced Materials for New Energy Storage and Conversion, Hunan University of Science and Technology, Xiangtan 411201, China; Institute of High Performance Computing, A*STAR, Singapore 138632, Singapore; Institute of High Performance Computing, A*STAR, Singapore 138632, Singapore; School of Mechanical and Aerospace Engineering, College of Engineering, Nanyang Technological University, Singapore 637457, Singapore

**Keywords:** low-buckled two-dimensional materials, strain effect, thermal conductivity, bonding dynamics

## Abstract

The thermal conductivity of two-dimensional materials, such as graphene, typically decreases when tensile strain is applied, which softens their phonon modes. Here, we report an anomalous strain effect on the thermal conductivity of monolayer silicene, a representative low-buckled two-dimensional (LB-2D) material. ReaxFF-based molecular dynamics simulations are performed to show that biaxially stretched monolayer silicene exhibits a remarkable increase in thermal conductivity, by as much as 10 times the freestanding value, with increasing applied strain in the range of [0, 0.1], which is attributed to increased contributions from long-wavelength phonons. A further increase in strain in the range of [0.11, 0.18] results in a plateau of the thermal conductivity in an oscillatory manner, governed by a unique dynamic bonding behavior under extreme loading. This anomalous effect reveals new physical insights into the thermal properties of LB-2D materials and may provide some guidelines for designing heat management and energy conversion devices based on such materials.

## Teaser

ReaxFF-based equilibrated molecular dynamics revealed an anomalous strain effect on the thermal transport of low-buckled two-dimensional silicene, which could provide useful guidelines for thermal manipulation in practical applications.

## INTRODUCTION

Since the first discovery of graphene, two-dimensional (2D) materials have drawn worldwide attention because of their distinguished mechanical, electrical and thermal properties, as well as applications in fields such as nanoelectronic, spintronic, valleytronic, thermoelectric, photovoltaic and optoelectronic devices [1–5]. The atomic-scale thickness of 2D materials can lead to a significant quantum confinement effect and new physical phenomena. For example, when the system size is smaller than the phonon de Broglie wavelength, the thermoelectric power factor greatly increases because of the sharper edge of the electron density of states, as theoretically predicted by Hicks and Dresselhaus [6] and recently demonstrated in monolayer InSe [7]. Moreover, non-Fourier thermal conductivity was discovered both theoretically and experimentally, resulting from the reduced phonon population and suppressed scattering rate [8–11]. In 2D materials, phonons are dominant carriers in thermal transport because of the low electron concentration [12]. Phonons also play important roles in other physical processes, e.g., phonon–electron coupling can renormalize the electron population and carrier dynamics [13,14].

The thermal conductivity of 2D materials is directly related to an array of applications, including advanced thermal management [15,16], thermal barrier coatings [17,18] and thermoelectrics [19]. In the first field, a high thermal conductivity is preferred, whereas for the latter two fields, a low thermal conductivity is desired. Hence, the thermal conductivity of 2D materials should be precisely controlled upon integration into devices. Monoatomic-layer films often suffer from prestraining during growth as well as integration [20], and strain engineering could serve as a powerful way to modulate the physical properties of 2D materials [21,22]. Thus, it is vital to understand the strain effect on the thermal conductivity of 2D materials.

From a geometrical point of view, 2D materials can be divided into two classes: flat materials, such as graphene, and low-buckled materials, such as silicene, germanene and stanene. The strain effect on the thermal conductivity of graphene has been studied by both classical molecular dynamics [23] and first-principles calculations [24], with the conclusion that a tensile strain decreases the thermal conductivity as a result of strain-induced softening of phonon modes. Compared with flat 2D materials, the corresponding strain effect on low-buckled 2D (LB-2D) materials remains largely unexplored.

In this work, we study the effect of biaxial tensile strain on the thermal conductivity of monolayer silicene, a representative LB-2D material that has been successfully synthesized, with promising applications in 2D field-effect transistors [25–28]. We report an anomalous two-stage strain effect in which the thermal conductivity increases by as much as 10 times with increasing applied strain in the range of [0, 0.1], and then plateaus in an oscillatory manner with a further increase in strain. This behavior will be rationalized by a combination of the phonon lifetime, atomic configuration and bonding characteristics.

## COMPARISON OF COMPUTATIONAL METHODS

The understanding of the effect of strain on the thermal conductivity of 2D materials has been limited by a lack of reliable experimental tools [2]. Because silicene is a semiconductor with a band gap of 1.55 meV [29], phonons are the dominant heat carriers, and the contribution from electrons should be negligible. The phonon Boltzmann transport equation (PBTE) and molecular dynamics (MD) simulation are the commonly used numerical methods in studying phonon-associated thermal conductivity. The PBTE with phonon properties extracted from first-principles calculations has been used to calculate the thermal conductivity of individual materials [30–32], as well as interface scattering in nanocomposites [33]. The solution of the PBTE is based on a statically optimized atomic configuration, and thus cannot provide physical insights about the dynamic characteristics discussed in the following sections. MD simulations include nonequilibrium molecular dynamics (NEMD) and equilibrium molecular dynamics (EMD). NEMD involves calculating the thermal conductivity based on the heat flux under a temperature gradient, and requires a sufficiently large sample size and a long simulation time. EMD is based on the Green-Kubo formula from the fluctuation-dissipation theorem and linear response theory. The accuracy of MD depends on the interatomic potentials. Each simulation method has its own advantages [34]. First-principles-based PBTE does not depend on the selection of empirical interatomic potentials, thus has high accuracy in predicting the absolute value of thermal conductivity. However, in this work, as bond dynamics plays a governing role that cannot be captured by PBTE, we selected MD to conduct our study.

The Tersoff and MEAM potentials have been used with NEMD to study the effect of uniaxial strain on the thermal conductivity of monolayer silicene, but these potentials were originally developed to describe interatomic interactions in bulk silicon rather than in atomically thin silicene, leading to inaccurate stress–strain relationships [35] and unsuccessful reproduction of the LB geometry [36]. In this regard, the Reax force field (ReaxFF) provides a better alternative [37,38]: On the one hand, it has been customized for silicene and can accurately characterize the LB geometry and material properties; on the other hand, it can meet the requirement of bond breaking and formation in a continuous way because of its preferred bond order. Therefore, in this work, we exploit ReaxFF to study the effect of biaxial strain on the thermal conductivity of monolayer silicene. As demonstrated in the following, the ReaxFF-based EMD reveals an anomalous strain effect on the thermal conductivity that has not been previously observed using the Tersoff or MEAM potential [35,36].

## RESULTS AND DISCUSSIONS

### The two-stage energetic response to strain

**Figure 1.**
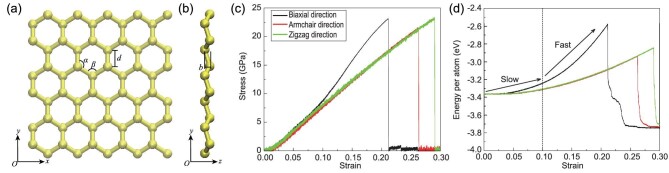
Schematic illustration of monolayer silicene from (a) the front view and (b) side view. (c) Stress–strain relationships and (d) energy–strain relationships of monolayer silicene under uniform biaxial tension and uniaxial tension in the armchair and zigzag directions.

Figure 1(a and b) illustrates the silicene structure optimized by ReaxFF, in which the bond length *d *= 0.23 nm, bond angles *α* = *β* = 112º and buckling distance *b *= 0.067 nm are all in good agreement with DFT calculations [39,40]. Figure 1(c and d) presents mechanical and energetic responses under biaxial tension and uniaxial tension along the armchair and zigzag directions, where the fracture strains are recorded as 0.21, 0.26 and 0.29, respectively. Included in the Supplementary data are snapshots of the deformation behavior around the fracture strain, showing the process of crack initiation and propagation. The uniaxial stiffnesses calculated from linear fitting of the stress–strain curves are 88.46 ± 0.04 GPa along the armchair direction and 87.83 ± 0.04 GPa along the zigzag direction, a within 1% disparity, indicating the high in-plane isotropy of silicene. Under biaxial tension, as the applied strain increases, the stress increment remains nearly constant, while the total energy (potential energy plus kinetic energy) increment becomes increasingly larger above strain *ϵ *≈* *0.1, as marked by the dashed line in Figure 1(d). This implies that atoms in silicene remain in a relatively stable state below strain *ϵ* ≈ 0.1 and then become increasingly active with a further increase in strain. To demonstrate the high degree of precision of ReaxFF, *ab initio* molecular dynamics (AIMD) was conducted to calculate the energy change under biaxial tension, with the results confirming the same two-stage energetic response as in our ReaxFF-based MD results (see Supplementary data). Meanwhile, the phonon dispersion of silicene is calculated from the first-principles as well as ReaxFF-based lattice dynamics (Supplementary data). Both the structural properties and the phonon spectrum calculated from ReaxFF agree well with those from the first-principles.

### Geometry evolution causes the thermal conductivity to increase under low strain

In Green-Kubo-based EMD, the thermal conductivity *κ* of a 2D material is calculated by integrating the heat current autocorrelation function (HCACF):

(1) \begin{equation*} \kappa = \frac{1}{{2{k_B}{T^2}V}}\int_{0}^{\infty }{{dt\left\langle {{{\bf S}}\left( 0 \right) \cdot {{\bf S}}\left( t \right)} \right\rangle}}, \end{equation*}

where *k_B_* is the Boltzmann constant, *T* is the temperature, *V* is the volume of the simulated sample, and **S** is the heat flux. The factor of 2 in the denominator ensures that the value is averaged over the two in-plane directions. A further discussion of this equation is briefly provided in the Supplementary data. Details of the simulation setup can be found in Methods. For integrating the HCACF, the cutoff time *τ*_c_ is carefully chosen based on the first dip (FD) method [41], corresponding to the moment when the tail of the HCACF first decays to zero. For comparison, we also tried an improved ‘first avalanche’ (FA) method [42] and obtained consistent results in both cases.

**Figure 2.**
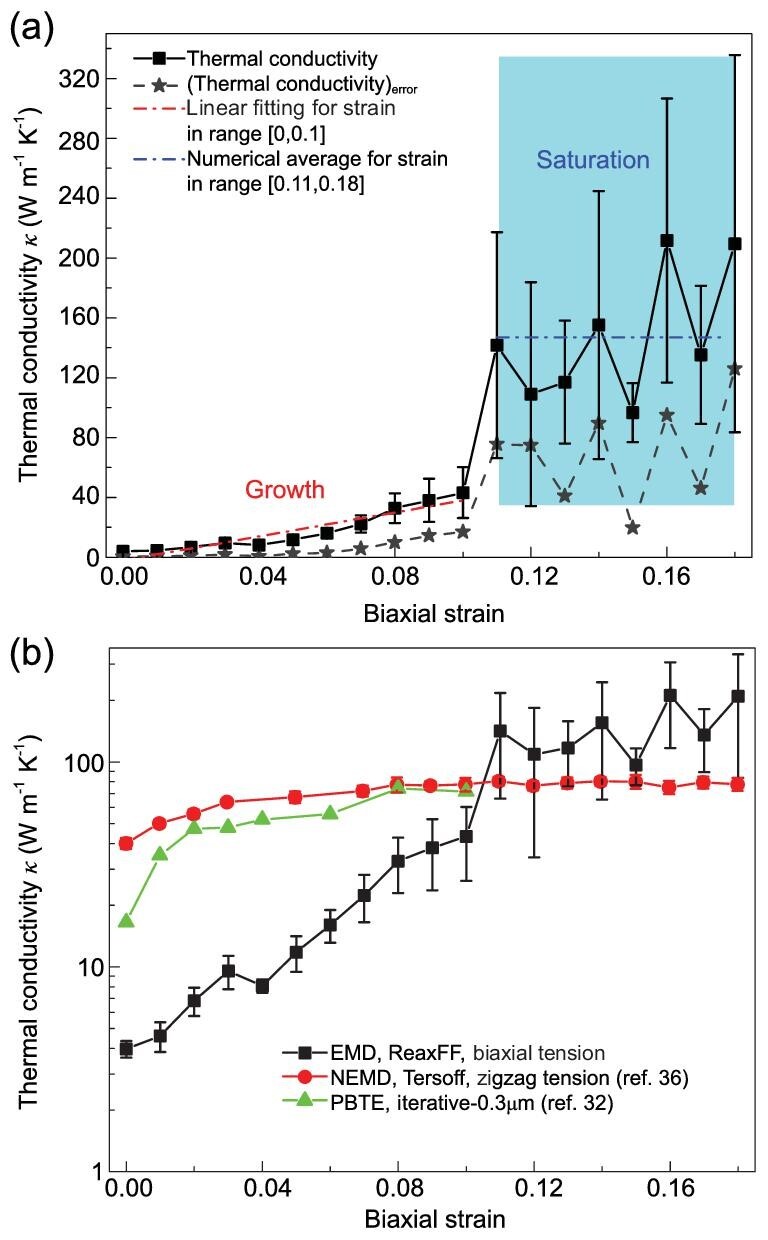
(a) Relationship between the applied biaxial strain and thermal conductivity of monolayer silicene. Each black square point is averaged over five repeated calculations with different initial velocity distributions. Gray pentagram points indicate standard errors. The red dashed line is the linear fit within the strain range of [0, 0.1], and the blue dashed line shows the average value within the strain range of [0.11, 0.18]. (b) Comparison with previous results obtained by the NEMD method (red) [36] and PBTE method (green) [32].

Figure 2(a) shows the relationship between the applied biaxial strain and thermal conductivity of monolayer silicene, where each black square point represents an average over five repeated calculations with different initial velocity distributions and gray pentagram points show standard errors. The thermal conductivity response to increasing biaxial strain shows a two-stage dependence that has not been previously reported. In the first stage, within the strain range of [0, 0.1], the thermal conductivity monotonically increases from 3.97 W m^−1^ K^−1^ at *ϵ *= 0 to 43.26 W m^−1^ K^−1^ at *ϵ *= 0.1, which is more than 10 times larger. In the second stage, within the strain range of [0.11, 0.18], as the strain further increases, the thermal conductivity starts to fluctuate within a range with an average of 147 W m^−1^ K^−1^, as shown by the blue area and blue line in Figure 2(a). Figure 2(b) compares our result (black line, ReaxFF-based EMD, biaxial tension) with results from a Tersoff-based NEMD study under uniaxial tension (red line) [36] and from a DFT-based PBTE study under biaxial tension (green line) [32]. The y-axis is set to a logarithmic scale to better show the difference. In the Tersoff-based NEMD study, as the uniaxial strain increases, the thermal conductivity first increases and quickly plateaus. However, as previously discussed, because the Tersoff potential fails to reproduce the initial LB geometry, it cannot be reliably used to calculate the strain-dependent geometry and thermal property evolution. In the DFT-based PBTE study, as the biaxial strain increases within the range of [0, 0.1], the thermal conductivity of silicene with a finite sample size (0.3 μm) continues to increase. Although the PBTE predicts similar results to our study, it is based on DFT and limited to static optimizations, while our study includes the bonding dynamics. Next, we will show how the geometry evolution and bonding dynamics affect the thermal responses.

**Figure 3.**
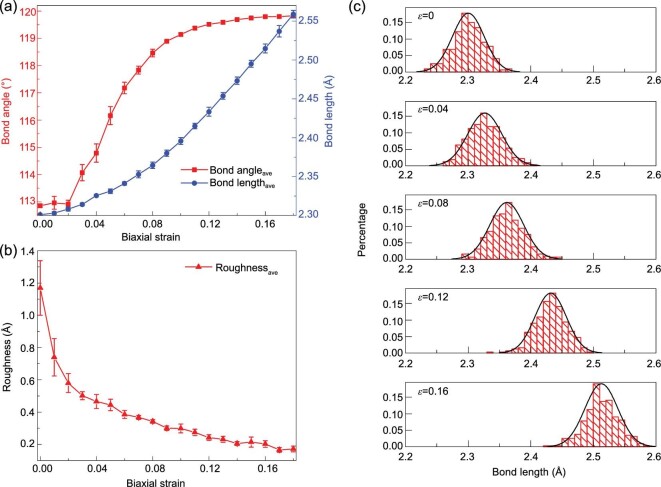
Geometry evolution in monolayer silicene under biaxial tension. (a) Bond angle, bond length and (b) roughness as functions of strain. These values are averaged over five calculations for each biaxial strain. (c) Bond length distributions under biaxial strains of 0, 0.04, 0.08, 0.12 and 0.16.

Usually, tensile strain softens the phonon modes, leading to a reduction in the in-plane lattice thermal conductivity of 2D materials [23]. To understand the unusual change in the thermal conductivity of monolayer silicene, we first analyzed the geometry evolution with increasing biaxial tension. Figure 3(a) depicts the variations in the average bond angle and bond length. At zero strain, the average bond angle and bond length are 112.86º and 2.30 Å, respectively. As the applied strain increases to *ϵ *= 0.1, the average bond angle increases to 119.14º, and the bond length increases to 2.39 Å. In the first stage, the deformation is reflected mainly through a flattening of the bond angle by 6.28º and an elongation of the bond length by 0.09 Å. In the second stage, as the applied strain further increases from 0.11 to 0.18, the average bond angle increases to 119.83º, and the bond length increases to 2.56 Å. Therefore, the tensile strain in the second stage elongates the bond length by 0.17 Å, almost twice that in the first stage, while the bond angle is flattened by 0.69º, almost 10 times smaller than that in the first stage. In addition, it is worth noting that even as the applied biaxial strain approaches the fracture strain (∼0.21, as shown in Figure 1(c)), the average bond angle approaches but never reaches 120°, which means that the LB geometry is an intrinsic property, as confirmed by a DFT study [43]. This underlines the importance of the LB geometry as a prerequisite for the choice of the interatomic potential.

Variations in the average roughness are tracked and plotted in Figure 3(b). Here, the roughness is calculated by the root mean square method, *R* = [(*Z*_1_^2 ^+ *Z*_2_^2^+$\cdots$+*Z*_N_^2^)/*N*]^1/2^. The value of *R* at zero strain is 1.16 Å, and it quickly decays as the biaxial strain increases, dropping by as much as 0.87 Å in the first stage (strain range of [0, 0.1]) and becoming 0.12 Å (almost seven times smaller) at the end of the second stage (strain range of [0.11, 0.18]). The variations in all three properties (bond angle, bond length and roughness) confirm a two-stage geometric response to biaxial strain. Figure 3(c) shows the local bond length distribution in the first stage (*ϵ *= 0, 0.04 and 0.08) and second stage (*ϵ *= 0.12 and 0.16). The black envelope curves show that the bond lengths obey the normal distribution *N*(*μ*, *σ*^2^) over the whole strain range, where *μ* is the mean and *σ* is the standard deviation.

The slight increase in the average bond length in the first stage (strain range of [0, 0.1]) should normally result in bond softening and a reduction in the thermal conductivity, contrary to the enhancement in the thermal conductivity observed in Figure 2. To understand this apparent anomaly, a double exponent fitting approach [44,45] with two time constants was utilized to fit the decay of the normalized HCACF Cor(t)/Cor(0). This concept was first proposed to differentiate the thermal transport contribution from fast optical modes and slow acoustic modes [45]. In physics, the decay of the HCACF results from the different relaxation times of short- and long-wavelength phonons. The fitting equation is expressed as

(2) \begin{equation*} \frac{{{\rm{Cor}}\left( t \right)}}{{{\rm{Cor}}\left( 0 \right)}} = {A_1}{e^{ - t/{\tau _1}}} + {A_2}{e^{ - t/{\tau _2}}}, \end{equation*}

in which *A_1_* and *A_2_* are fitting parameters, and *τ_1_* and *τ_2_* are the relaxation times of short- and long-wavelength phonons.

**Figure 4.**
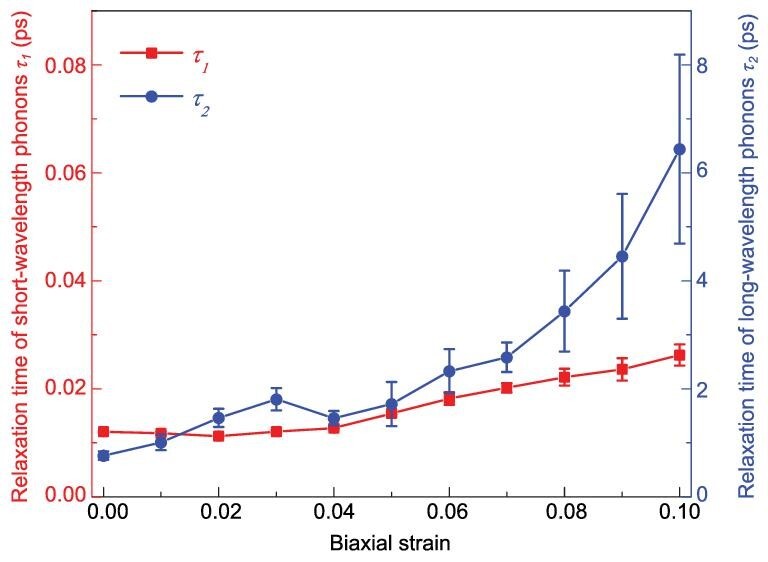
Relaxation times of short- and long-wavelength phonons within the strain range of [0, 0.1].

Figure 4 shows the variations in the relaxation times of short- and long-wavelength phonons with biaxial strain. As the applied strain rises from 0 to 0.1, the relaxation time of long-wavelength phonons *τ*_2_ increases from 0.76 ps to 6.44 ps, by approximately eight times, while the relaxation time of short-wavelength phonons *τ*_1_ increases from 0.012 ps to 0.026 ps, which is more than twice as large. Within the biaxial strain range of [0, 0.1], the observed flattening of the LB geometry leads to an increased contribution from long-wavelength phonons, which prevails over the negative impact induced by the slightly elongated bond length. Therefore, under relatively low strain, the increased thermal conductivity of silicene is a consequence of the 8-fold enhanced lifetime of long-wavelength phonons, which benefits from the flattened LB geometry.

### Bonding dynamics causes the thermal conductivity to saturate at high strain

**Figure 5.**
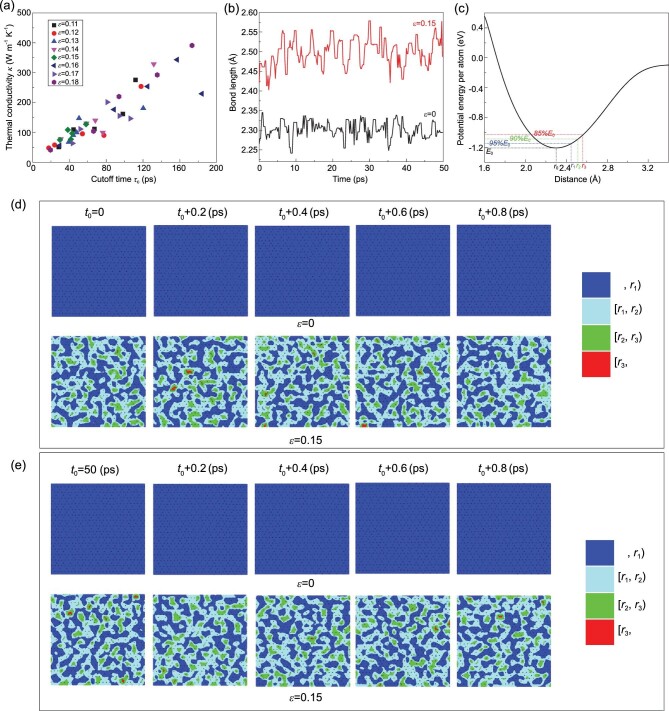
(a) Thermal conductivity versus cutoff time within the strain range of [0.11, 0.18]. Points with the same shape and color indicate the same biaxial strain condition. (b) Bond length variation of a randomly selected bond from silicene samples under two biaxial strains (*ϵ *= 0 and 0.15) during the heat flux collection period. (c) Potential energy per atom calculated by ReaxFF as a function of the distance between two silicon atoms in an isolated system. The inset shows the calculation system schematic. (d and e) Contour evolution of the bond length distribution in silicene samples under two biaxial strains (*ϵ *= 0 and 0.15) within a short period starting from (d) *t*_0_ = 0 and (e) *t*_0_ = 50 ps. The black points in the contours represent the centers of Si-Si bonds. The blue, cyan, green and red colors represent bond lengths smaller than *r*_1_, in the range of [*r*_1_, *r*_2_), in the range of [*r*_2_, *r*_3_) and larger than *r*_3_. The upper and lower bounds of each color (*r*_1_, *r*_2_, *r*_3_) classify different degrees of bond strength as extracted from (c).

In the observed anomalous strain effect on the thermal conductivity *κ *of silicene, a unique behavior is the saturation of *κ* in the strain range of [0.11, 0.18]. To understand why the thermal conductivity stops increasing in this stage, we analyzed the relationship between *κ* and cutoff time *τ*_c_, defined as the time at which the HCACF decays to 0 (Figure 5(a)). Detailed in the Supplementary data are five calculations of the HCACF under biaxial strains of *ϵ *= 0.12, 0.14, 0.16 and 0.18. In the fluctuation-dissipation theorem and linear response theory, the time correlation function describes the dynamics of the whole system. In principle, for samples under different strains, the cutoff time is different for different thermodynamic states, leading to different decay rates. For different realizations under the same strain, although different initial velocity distributions may not result in equal decay rates, the cutoff times should only vary in a small range. However, as shown in Figure 5(a), points with the same color and shape (meaning the same strain condition) are scattered in space rather than concentrated in a small region, which leads to the fluctuation in the thermal conductivity under high biaxial strain.

Furthermore, from the point of bonding dynamics, we compared the bond length variation of a randomly selected bond from silicene samples under biaxial strains of *ϵ *= 0 and *ϵ *= 0.15 during heat flux collection, as shown in Figure 5(b). The length

 

variations of the two bonds are sinusoidal like, with fluctuation amplitudes of 0.06 Å at *ϵ *= 0 and 0.18 Å at *ϵ *= 0.15, which means that local bonds tend to elongate and shorten with larger amplitude under higher strain. To see how the enhanced bonding dynamics affects interatomic interactions, an isolated system with only two silicon atoms was constructed, as schematically illustrated by the inset in Figure 5(c). In this system, one silicon atom is fixed while the other is moved away. The interaction energy between the two atoms as a function of distance was measured by the ReaxFF approach used in this study and plotted in Figure 5(c). The most stable distance between two silicon atoms is *r*_0_ = 2.30 Å, the same as the equilibrated Si-Si bond length in silicene, with a minimum energy of *E*_0_ = −1.21 eV. We identified the interatomic distances corresponding to 95%*E*_0_, 90%*E*_0_ and 85%*E*_0_ as *r*_1_ = 2.45 Å, *r*_2_ = 2.52 Å and *r*_3_ = 2.57 Å. From a global perspective, as shown in Figure 3(a), when the biaxial strain reaches 0.18, the average bond length is 2.56 Å, which is still smaller than *r_3_* corresponding to 85%*E_0_*. This suggests that there is no significant bond weakening in silicene within the strain range under investigation, consistent with the brittle stress–strain relationships shown in Figure 1(c). Furthermore, we used *r*_1_, *r*_2_ and *r*_3_ to classify the bond lengths (the degrees of bond strength) in the whole silicene sample. Figure 5(d and e) depicts the contour evolution of the bond length distribution in silicene samples under two typical biaxial strains (*ϵ *= 0 and 0.15) within a short period starting from two different moments *t*_0_. Here, the black points mark the centers of each Si-Si bond, and the different colors represent different bond length ranges bounded by *r*_1_, *r*_2_ and *r*_3_. In a short period starting from *t*_0_ = 0, as shown in Figure 5(d), every bond in the silicene sample under strain *ϵ *= 0 (the first row) stays within the range of *r_1_*, while under strain *ϵ *= 0.15 (the second row), the bonds experience rapid oscillations everywhere. This phenomenon also exists during the period starting from *t*_0_ = 50 ps, as shown in Figure 5(e), where all Si-Si bonds in silicene stay stable within the same range (blue) under strain *ϵ *= 0 (the first row) while they continuously shuttle through different typical ranges (cyan, green and red) under strain *ϵ *= 0.15 (the second row).

According to these two randomly selected short periods, it can be speculated that under high strain, silicene attains a unique dynamic equilibrium in which atoms are more active and bonds ceaselessly elongate and shorten in a moderate manner. In this kind of equilibrium, the bonds are subject to more length fluctuations with strong perturbations to the interatomic potential [46]. From Klemens’ perturbation theory [47], the rate of phonon scattering for different force constants is given as $\Gamma \propto {\delta ^2}$, where δ is the change in the force constant. Therefore, the large fluctuations in the local bond lengths under extreme tensile loading can generate additional phonon scattering. Overall, the flattened LB geometry suppresses the scattering of long-wavelength phonons, while the enhanced bond dynamics introduces extra phonon scatterings. The competition between these two mechanisms leads to a plateau in the thermal conductivity.

## CONCLUSIONS

In conclusion, by applying ReaxFF-based EMD, we have revealed an anomalous strain effect on the thermal conductivity of monolayer silicene, a representative LB-2D material. ReaxFF is chosen to provide an effective and accurate description of both the LB geometry and bonding dynamics. We find that the thermal conductivity of silicene increases within the strain range of [0, 0.1] and saturates to a plateau within the strain range of [0.11, 0.18]. The first stage of increasing thermal conductivity is due to strain-induced flattening of the LB-2D geometry, which enhances the thermal transport contribution from long-wavelength phonons. The second stage of saturation of the thermal conductivity is a consequence of the competition between the continuously flattened geometry and increasing bond length fluctuation at high strain. Our results not only reveal an anomalous effect of biaxial strain on the thermal conductivity of monolayer silicene but also may suggest a general principle applicable to all LB-2D materials. These findings significantly enrich our fundamental understanding of the strain effects on heat conduction in 2D materials and provide useful guidelines for manipulating the thermal conductivity in practical applications. Finally, we would like to mention that at present, it is still a challenge to separate silicene from a substrate. In this work, we focus on a universal strain effect on the in-plane thermal conductivity. If the interaction between silicene and substrate is sufficiently weak, such as in the case of van der Waals interaction, the phenomena reported in the present work are expected to be observable.

## METHODS

All of our ReaxFF-based EMD simulations were performed with a large-scale atomic/molecular massively parallel simulator (LAMMPS) [48,49]. The thickness of silicene is 4.2 Å. Periodic boundary conditions were applied in the two in-plane directions, and the in-plane size of the simulation box was chosen to be 6 nm, beyond which the thermal conductivity result becomes size-independent (see Supplementary data). The integration time step was 0.2 fs, the same as in previous ReaxFF-based interface studies [50]. The sample was first equilibrated at room temperature (300 K) and zero pressure for 5 × 10^5^ steps before a biaxial strain was applied by stretching the box at a constant strain rate of 1e^−6^ per fs until the desired strain was reached. The strain level was stepwise increased in the range between 0 and 0.18 at an interval of 0.01. At each level, to eliminate the possible influence of the strain rate, after the desired strain was reached, the silicene sample was relaxed under the NVT ensemble (fixed volume and room temperature via a Nose-Hover thermostat) for another 2.5 × 10^6^ steps to attain full equilibrium. Subsequently, the ensemble was changed to NVE to collect the heat flux of each loading step for 3 × 10^6^ steps.

## Supplementary Material

nwaa220_Supplemental_File
