# Supplementary material for: Anomalous strain effect on the thermal conductivity of low-buckled two-dimensional silicene
Source: Natl Sci Rev. 2020 Aug 31;8(9):nwaa220. doi: 10.1093/nsr/nwaa220 (PMC8433080; doi:10.1093/nsr/nwaa220)
Supplement: nwaa220_Supplemental_File [file nwaa220_supplemental_file.docx]

**Anomalous strain effect on the thermal conductivity of low-buckled two-dimensional silicene**

Bin Ding^1^, Xiaoyan Li^2^, Wuxing Zhou^3^, Gang Zhang^1,*^, Huajian Gao^1,4,*^

^1^Institute of High Performance Computing, A*STAR, Singapore 138632, Singapore

^2^Centre for Advanced Mechanics and Materials, Applied Mechanics Laboratory, Department of Engineering Mechanics, Tsinghua University, Beijing 100084, China

^3^School of Materials Science and Engineering & Hunan Provincial Key Laboratory of Advanced Materials for New Energy Storage and Conversion, Hunan University of Science and Technology, Xiangtan, 411201, China

^4^School of Mechanical and Aerospace Engineering, College of Engineering, Nanyang Technological University, 70 Nanyang Drive, Singapore 637457, Singapore.

^*^Corresponding Author. E-mail: [zhangg@ihpc.a-star.edu.sg](mailto:zhangg@ihpc.a-star.edu.sg), [huajian.gao@ntu.edu.sg](mailto:huajian.gao@ntu.edu.sg)

**S1. Crack initiation and fast propagation in monolayer silicene under biaxial tension and uniaxial tension along the armchair and zigzag directions**

**
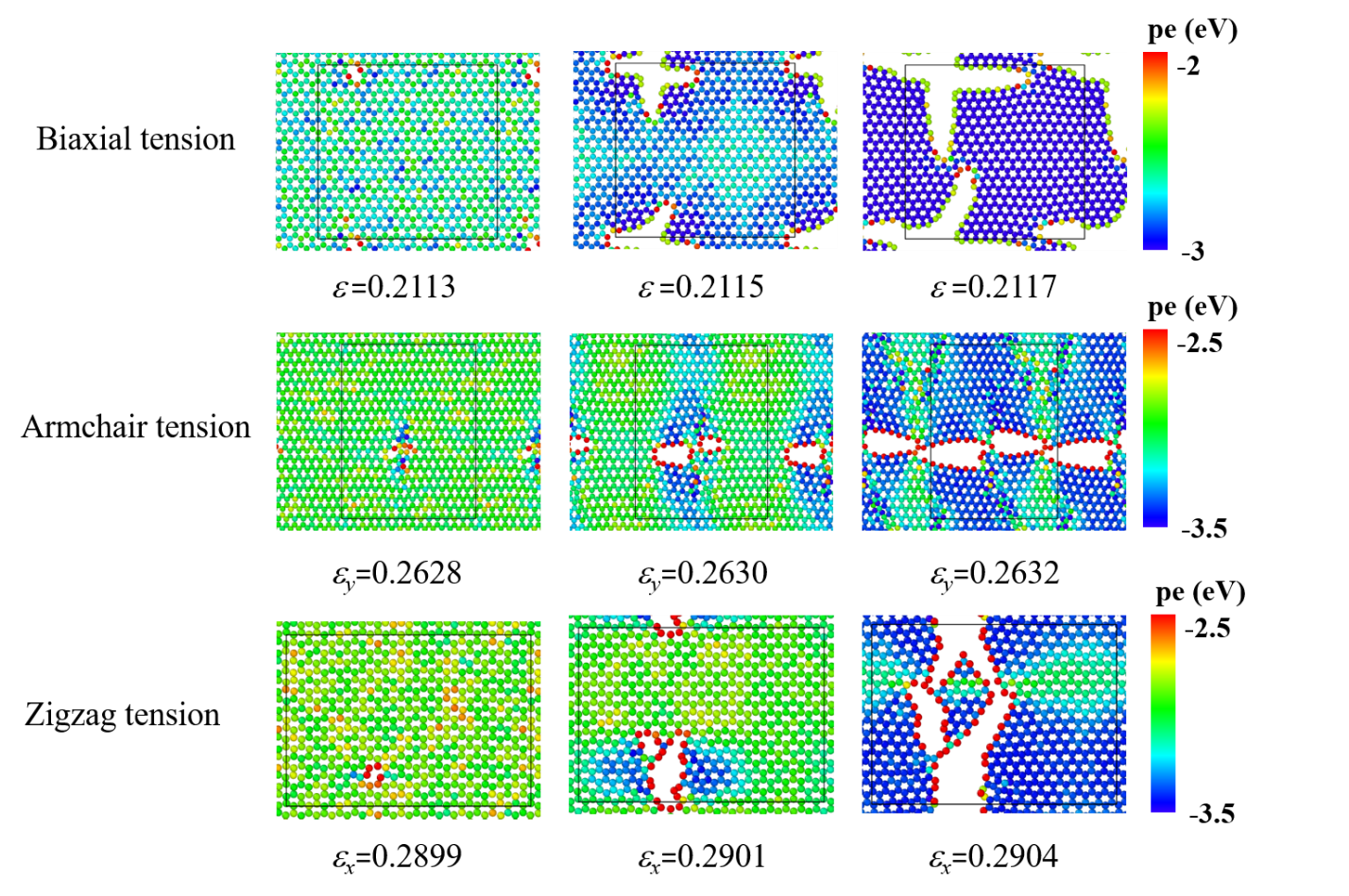
**

**Figure S1.** Deformation and failure behaviors of monolayer silicene under biaxial tension as well as uniaxial tension along the armchair and zigzag directions. Atoms are colored by potential energy. For all three loading conditions, once the fracture strain is reached, cracks initiate and quickly run through the whole sample.

**S2. *Ab initio* molecular dynamics simulations**

We performed *ab initio* molecular dynamics (AIMD) simulations by VASP[1] to calculate the energy change of monolayer silicene during biaxial tension. A 6 × 6 × 1 supercell is built as our model which contains 72 atoms. The temperature is maintained at room temperature (T=300 K) via NVT ensemble. A 1 × 1 × 1 Gamma centered k-point[2] is adopted. Time step was chosen as 1 fs and energy was collected after 1,500 steps of equilibrium process. The energy cutoff and a total energy tolerance were set as 375 eV and 10^-6^ eV, respectively. The projector augmented wave (PAW)[3] method and the local density approximation (LDA)[4] functional were applied to calculate electron-ion interactions and exchange-correlation interactions. Figure S2 shows energy change of monolayer silicene during biaxial tension from AIMD simulations. There also exists an obvious turning point around *ε*=0.1, before which atoms are relatively stable and after which atoms become more active. Energy change per atom from *ε*=0 to *ε*=0.1 is 0.16 eV based on AIMD simulations, which is close to that (0.12 eV) from ReaxFF-based MD simulations. Energy change per atom from *ε*=0.1 to *ε*=0.18 is up to 0.43 eV from AIMD simulations, which is close to that (0.40 eV) from ReaxFF-based MD simulations. It indicates that the energy change results from AIMD are consistent with those from ReaxFF-based MD.

**
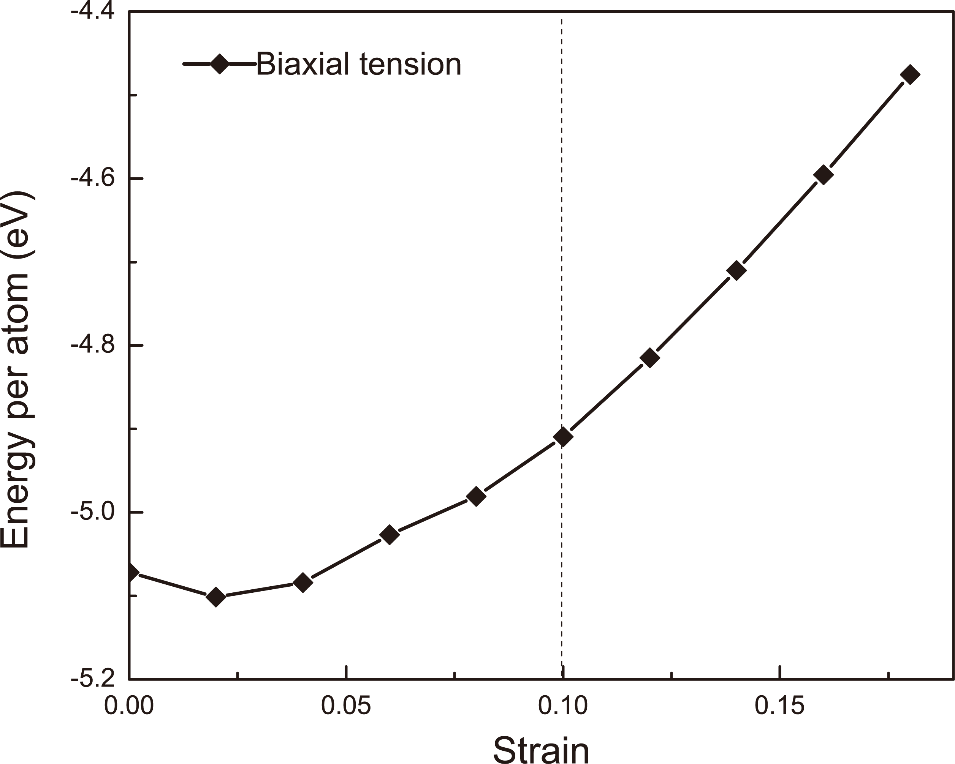
**

**Figure S2.** Energy change during biaxial tension based on AIMD method.

**S3. Phonon dispersion of silicene**

**
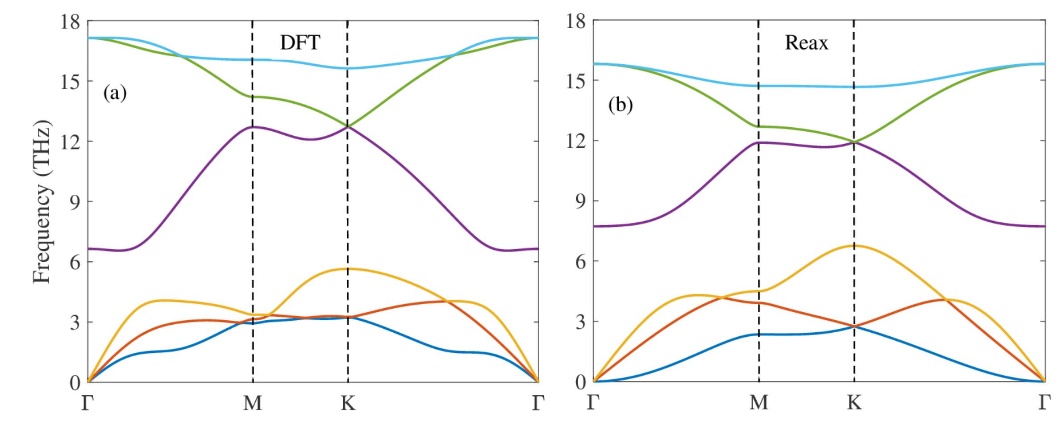
**

**Figure S3.** Phonon dispersion of silicene calculated from (a) the first-principles and (b) ReaxFF-based MD.

**S4. Thermal conductivity calculations in EMD**

According to the equilibrium molecular dynamics (EMD), the thermal conductivity is given by,

$\kappa_{\mu\nu}=\frac{1}{k_{B}T^{2}V}\int_{0}^{\infty} dt\left\langle S_{\mu}\left(0\right)S_{\nu}\left(t\right)\right\rangle$ (1)

where *μ* and *v* stand for the spatial directions. For mono-atomic layer silicene, thermal conductivity is averaged in in-plane direction and expressed as,

$\kappa=\frac{1}{2k_{B}T^{2}V}\int_{0}^{\infty} dt\left\langle\mathbf{S}\left(0\right)\cdot\mathbf{S}\left(t\right)\right\rangle$ (2)

The heat flux in Green-Kubo formalism is defined as,

$S=\frac{\text{d}}{\text{d}t}\sum_{i} \mathbf{r}_{i}\left(t\right)\varepsilon_{i}\left(t\right)$ (3)

where **r**_i_(t) and *ε*_i_(t) represent coordinate vector and total energy of atom *i* at time *t*, respectively. For multi-body interaction, the heat flux could be expanded as,

$S=\sum_{i} \mathbf{v}_{i}\varepsilon_{i}+\frac{1}{2}\sum_{\begin{matrix} i,j \\ i\neq j \end{matrix}} \mathbf{r}_{ij}\left(\mathbf{F}_{ij}\cdot\mathbf{v}_{i}\right)+\frac{1}{6}\sum_{\begin{matrix} i,j,k \\ i\neq j,i\neq k \end{matrix}} \left(\mathbf{r}_{ij}+\mathbf{r}_{ik}\right)\left(\mathbf{F}_{ijk}\cdot\mathbf{v}_{i}\right)$ (4)

where **v**_i_ represent the velocity of atom *i* ; **F**_ij_ and **F**_ijk_ represent two-body and three-body interaction, respectively.

**S5. Normalized heat current auto-correlation function (HCACF) under different biaxial strains**

**
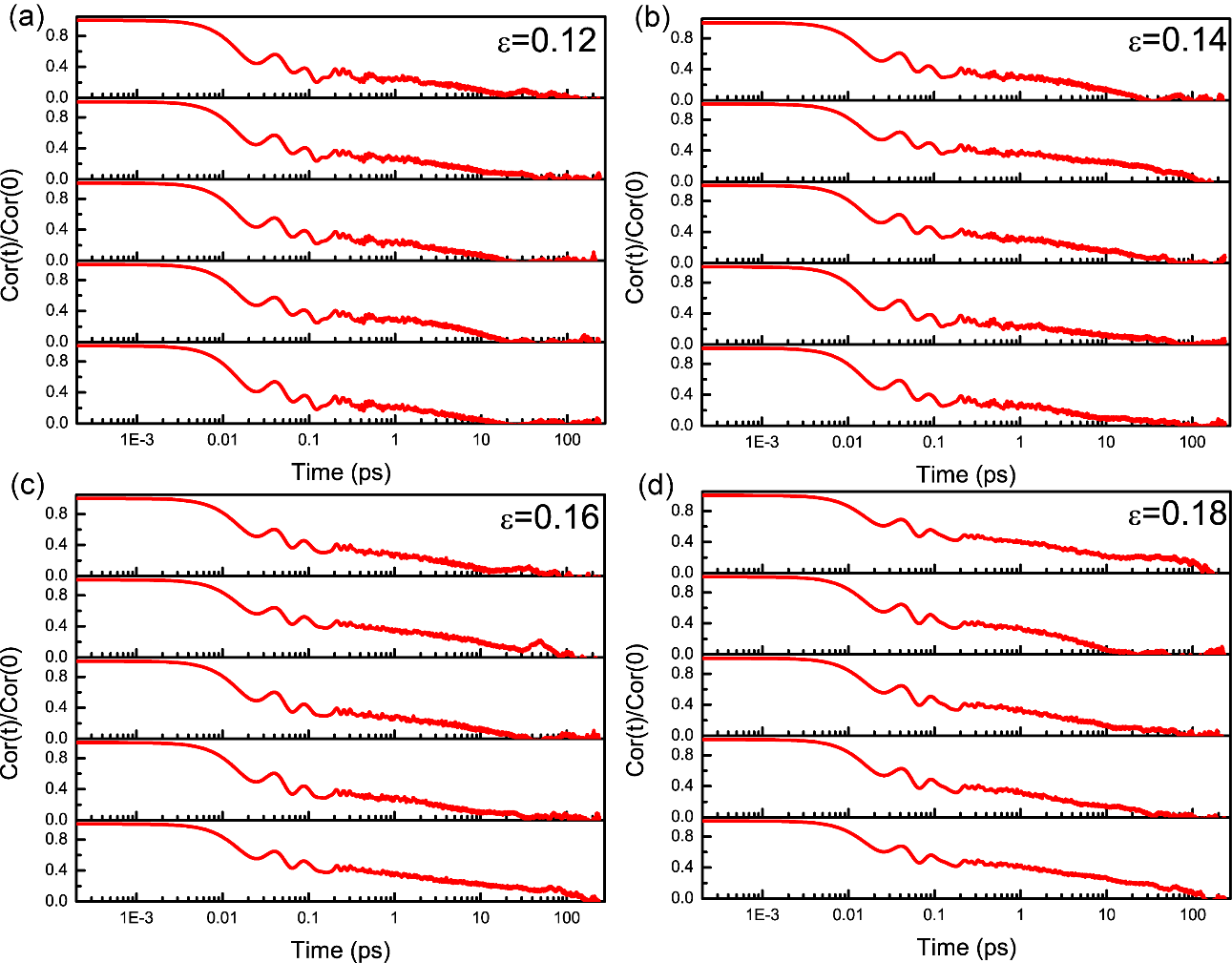
**

**Figure S4.** Normalized HCACF under biaxial strains of 0.12, 0.14, 0.16, 0.18.

**S6. Size dependence of the thermal conductivity calculation of monolayer silicene**

**
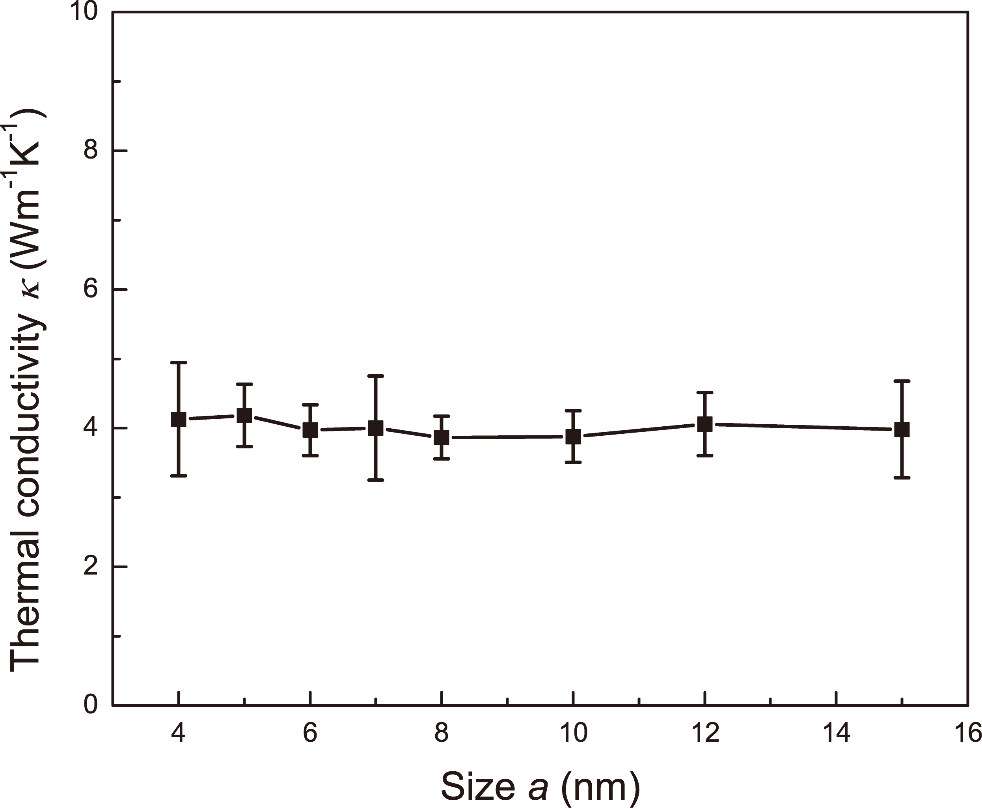
**

**Figure S5.** Relationship between sample size *a* and thermal conductivity value *κ*. Each point is averaged from five calculations with same sample size but different initial velocity distributions. The thermal conductivity remains nearly constant around 4 Wm^-1^K^-1^ under room temperature and zero pressure.

**REFERENCES**

1. Kresse G and Furthmüller J. Efficiency of ab-initio total energy calculations for metals and semiconductors using a plane-wave basis set. *Computational materials science*. 1996; **6**(1): 15-50.

2. Monkhorst HJ and Pack JD. Special points for Brillouin-zone integrations. *Phys Rev B*. 1976; **13**(12): 5188.

3. Blöchl PE. Projector augmented-wave method. *Phys Rev B*. 1994; **50**(24): 17953.

4. Perdew JP and Zunger A. Self-interaction correction to density-functional approximations for many-electron systems. *Phys Rev B*. 1981; **23**(10): 5048.
